# SOLTI-1805 TOT-HER3 Study Concept: A Window-of-Opportunity Trial of Patritumab Deruxtecan, a HER3 Directed Antibody Drug Conjugate, in Patients With Early Breast Cancer

**DOI:** 10.3389/fonc.2021.638482

**Published:** 2021-04-23

**Authors:** Tomás Pascual, Mafalda Oliveira, Eva Ciruelos, Meritxell Bellet Ezquerra, Cristina Saura, Joaquin Gavilá, Sonia Pernas, Montserrat Muñoz, Maria J. Vidal, Mireia Margelí Vila, Juan M. Cejalvo, Blanca González-Farré, Martin Espinosa-Bravo, Josefina Cruz, Francisco Javier Salvador-Bofill, Juan Antonio Guerra, Ana María Luna Barrera, Miriam Arumi de Dios, Stephen Esker, Pang-Dian Fan, Olga Martínez-Sáez, Guillermo Villacampa, Laia Paré, Juan M. Ferrero-Cafiero, Patricia Villagrasa, Aleix Prat

**Affiliations:** ^1^SOLTI Innovative Cancer Research, Barcelona, Spain; ^2^Medical Oncology Department, Hospital Clinic de Barcelona, Barcelona, Spain; ^3^Translational Genomics and Targeted Therapies in Solid Tumors, August Pi i Sunyer Biomedical Research Institute (Instituto de Investigaciones Biomédicas August Pi i Sunyer), Barcelona, Spain; ^4^Medical Oncology Department, Vall d'Hebron Institute of Oncology (VHIO), Hospital Universitari Vall d'Hebron, Vall d'Hebron Barcelona Hospital, Barcelona, Spain; ^5^Breast Cancer Program, Vall d'Hebron Institute of Oncology (VHIO), Hospital Universitari Vall d'Hebron, Vall d'Hebron Barcelona Hospital, Barcelona, Spain; ^6^Medical Oncology Department, Hospital 12 de Octubre, Madrid, Spain; ^7^Medical Oncology Department, IVO—Fundación Instituto Valenciano de Oncología, Valencia, Spain; ^8^Medical Oncology Department, Institut Catala d' Oncologia (ICO), H. U. Bellvitge-Institut d'Investigació Biomèdica de Bellvitge, Barcelona, Spain; ^9^Medical Oncology Department, ICO—Institut Català d' Oncologia Badalona, Hospital Universitario Germans Trias i Pujol, Badalona, Spain; ^10^Medical Oncology Department, Hospital Clínico Universitario de Valencia, Valencia, Spain; ^11^Breast Cancer Biology Research Group, Biomedical Research Institute Fundación para la Investigación del Hospital Clínico de la Comunidad Valenciana, Valencia, Spain; ^12^Pathology Department, Hospital Clinic of Barcelona, Barcelona, Spain; ^13^Breast Cancer Surgical Unit, Vall d' Hebron University Hospital, Barcelona, Spain; ^14^Medical Oncology Department, Hospital Universitario de Canarias, Santa Cruz de Tenerife, Spain; ^15^Medical Oncology Department, Hospital Universitario Virgen del Rocio, Sevilla, Spain; ^16^Medical Oncology Department, Hospital Universitario de Fuenlabrada, Madrid, Spain; ^17^Medical Oncology Department, Centro Integral Oncológico Clara Campal, Madrid, Spain; ^18^Research and Development, Daiichi Sankyo, Inc., Basking Ridge, NJ, United States; ^19^Oncology Data Science, Vall d'Hebron Institute of Oncology (VHIO), Barcelona, Spain; ^20^Medicine Department, University of Barcelona, Barcelona, Spain

**Keywords:** Breast Cancer, ERBB3, HER3, U3-1402, patritumab deruxtecan, HER3-DXd, CelTIL Score

## Abstract

**Background:** Preclinical data support a key role for the human epidermal growth factor receptor 3 (HER3) pathway in hormone receptor (HR)–positive breast cancer. Recently, new HER3 directed antibody drug conjugates have shown activity in breast cancer. Given the need to better understand the molecular biology, tumor microenvironment, and mechanisms of drug resistance in breast cancer, we designed this window-of-opportunity study with the HER3 directed antibody drug conjugate patritumab deruxtecan (HER3-DXd; U3-1402).

**Trial Design:** Based on these data, a prospective, multicenter, single-arm, window-of-opportunity study was designed to evaluate the biological effect of patritumab deruxtecan in the treatment of naïve patients with HR-positive/HER2-negative early breast cancer whose primary tumors are ≥1 cm by ultrasound evaluation. Patients will be enrolled in four cohorts according to the mRNA-based ERBB3 expression by central assessment. The primary endpoint is a CelTIL score after one single dose. A translational research plan is also included to provide biological information and to evaluate secondary and exploratory objectives of the study.

**Trial Registration Number:** EudraCT 2019-004964-23; NCT number: NCT04610528.

## Introduction

HER3, encoded by the *ERBB3* gene, is broadly expressed in various types of human cancer. HER3 has been associated with poor patient outcomes (1) and therapeutic agent resistance, including resistance to anti-EGFR, anti-HER2 inhibitors (2), and endocrine therapy (3, 4). HER3 belongs to the type I transmembrane tyrosine kinase family of receptors and activates intracellular signaling pathways, mainly the PI3K/AKT and MAPK/ERK pathways, upon dimerization with other HER family members (2, 5). These observations have resulted in the development of investigational HER3 directed agents in HER3-expressing breast cancer and other solid tumors.

Patritumab deruxtecan (HER3-DXd; U3-1402), a potential first-in-class HER3 directed antibody drug conjugate (ADC), is currently under development to act on these previously mentioned targets (6). In addition to its antitumor efficacy by binding HER3 ligand and the release of the cytotoxic payload in the tumor cells (7), patritumab deruxtecan enhanced the infiltration of innate and adaptive immune cells in preclinical models (8). These preclinical data have shown that patritumab deruxtecan can elicit potent antitumor immunity even in the setting of tumors insensitive to PD-1 and PD-L1 immune checkpoint inhibitors and that its efficacy is more pronounced in the presence of PD-1 inhibition, suggesting that patritumab deruxtecan sensitizes insensitive tumors to PD-1 blockade and has synergistic effects (8).

In the clinical setting, an early report of a clinical trial suggested that patritumab deruxtecan could be safely administered and it demonstrated promising antitumor efficacy (the overall response and the disease control rate were 42.9 and 90.5%, respectively) in heavily pretreated HER3-expressing metastatic breast cancer (9); these results are in accordance with more recent preliminary data from heavily pretreated EGFR-mutated non-small cell lung carcinoma patients, in whom the overall response rate was 25%, and the disease control rate was 70% (10).

Although no validated HER3 assay has been established to date, recent studies support the role of HER3 immunohistochemistry (IHC) as a potential biomarker (11–13). However, there are important limitations with IHC-based assays, such as different sensitivities of the antibodies used, their low dynamic range, their subjectivity in scoring, and their difficulty in establishing suitable cut-offs. Therefore, clinical implementation of a robust genomic assay would represent an important advancement. To overcome these limitations, we plan to test the prospective use of an mRNA-based *ERBB3* expression assay using the nCounter platform (Nanostring Technologies, Seattle, USA) developed by our group (14).

The role of the host immune system in breast cancer is becoming an important topic to study for several reasons. First, the immune response has a fundamental role in the efficacy of drug therapy. In all breast cancer subtypes, baseline high TIL grade is associated with a significantly higher pCR rate after neoadjuvant chemotherapy (15). Second, the recent success of therapeutic agents capable of activating immune responses to cancer, such as anti-PD1/PDL1 or anti-CTLA4 inhibitors, allows innovative treatment strategies (16). Third, high tumor-infiltrating lymphocytes (TILs) counts and immune-related gene expression signatures in the primary tumor are consistently associated with better survival in triple-negative breast cancer and HER2-positive breast cancer (15, 17–19). On the other hand, the prognostic value of assessing TILs in HR-positive/HER2-negative breast cancer remains unclear according to a few studies (15, 20).

The TOT-HER3 (a window-of-opportunity study of patritumab deruxtecan, a HER3 directed ADC in operable breast cancer according to *ERBB3* expression) trial is designed to assess whether a single dose of patritumab deruxtecan can increase immune infiltration and the lysis of tumor cells during short-term preoperative treatment in hormone receptor (HR)-positive/HER2-negative primary breast cancer. Short-term preoperative studies are a validated strategy for evaluating the impact of targeted therapies using the decrease in tumor cellularity and the increase in immune infiltration as a surrogate endpoint of treatment benefit (21, 22). The primary endpoint of TOT-HER3 is changes in the CelTIL score, a novel combined biomarker based on stromal TILs and tumor cellularity. Access to tumor tissue before and after the investigational treatment enables comprehensive analysis of biomarker changes, thus providing critical insights into the optimal patient population, biomarker predictive value, and potential mechanisms of primary resistance (23, 24).

## Methods

### Study Design and Treatment

**Figure 1:**
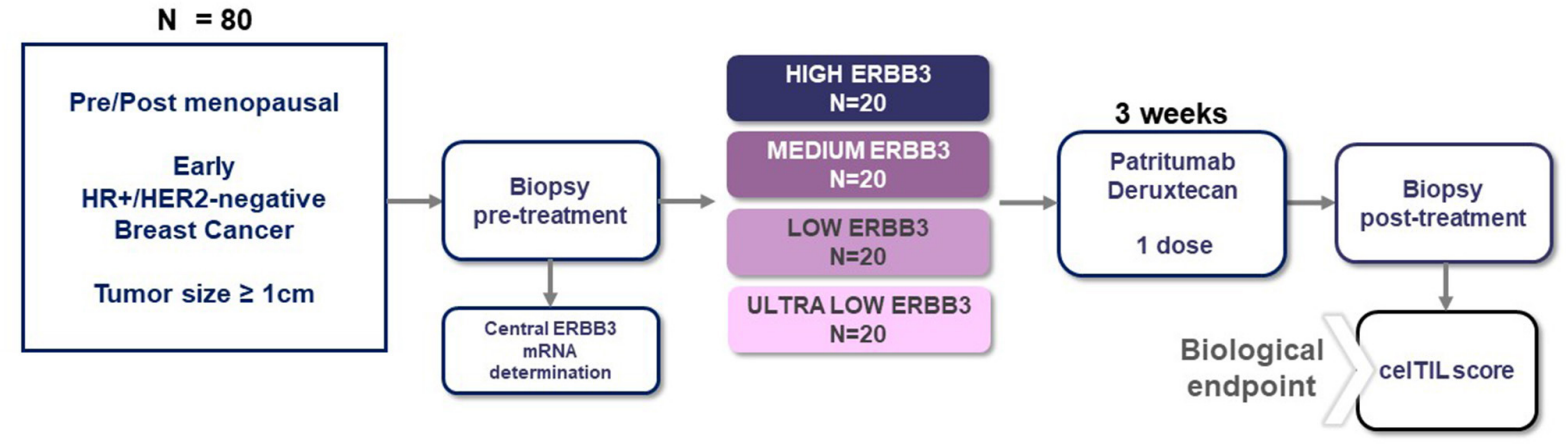
TOT-HER3 trial design.

This is a prospective, multicenter, single-arm, window-of-opportunity study evaluating the biological effect of patritumab deruxtecan in treatment naïve patients with early breast cancer, whose primary tumors are ≥1 cm by ultrasound evaluation (Figure 1). The study will include up to 80 patients with HR-positive/HER2-negative tumors.

Adult female patients (≥18 years old) with pre/post-menopausal status will be eligible if they have not been previously treated and have histologically confirmed stage I–IIIA invasive breast cancer, with primary tumors equal to or larger than 1 cm in diameter (as measured by ultrasound), clinical nodal status of 0–2, HR-positive and HER2-negative according to ASCO/CAP guidelines, and Ki67% ≥ 10% determined locally. Patients should also have an Eastern Cooperative Oncology Group (ECOG) performance status of 0–1 and adequate hematological counts, hepatic and renal function, and left ventricular ejection fraction ≥ 50%. Patients will be excluded if they have received prior anticancer therapy. Detailed inclusion and exclusion criteria can be found in Table 1.

**Table 1:** Main/key eligibility criteria.

**Inclusion Criteria**	**Exclusion criteria**
1. Written informed consent form. 2. Premenopausal or post-menopausal women and men, age ≥ 18 years. 3. ECOG Performance Status 0–1. 4. Histologically confirmed non-metastatic primary invasive adenocarcinoma of the breast untreated and recently diagnosed, with all the following characteristics: - At least one lesion that can be measured in at least 1 dimension with ≥ 1 cm in the largest diameter measured by ultrasound. - Absence of distant metastasis (M0) as determined by institutional practice. - in the case of a multifocal or multicentric tumor, the largest lesion must be ≥1 cm and designated the “target” lesion for all subsequent tumor evaluations and biopsies. 5. Patient must have biopsiable disease. 6. Estrogen (ER)-positive and/or Progesterone (PgR)-positive and HER2-negative tumor by the most recent American Society of Clinical Oncology—College of American Pathologists (ASCO-CAP) guidelines: ER and PgR defined as IHC nuclear staining >1% and HER2 negative locally assessed. 7. Ki67% ≥ 10% locally assessed. 8. Available pretreatment FFPE core needle biopsy evaluable for PAM50 and *ERBB3* mRNA expression. 9. Baseline LVEF ≥ 50% 10. Adequate organ functions 11. Absence of any psychological, familial, sociological, or geographical condition potentially hampering compliance with the study protocol and follow-up schedule; those conditions should be discussed with the patient before registration in the trial.	1. Inoperable locally advanced or inflammatory (i.e., inoperable stage III) breast cancer. 2. Bilateral invasive breast cancer. 3. Patients in whom a primary tumor excisional biopsy was performed. 4. Any prior treatment for primary actual invasive breast cancer. 5. Prior treatment with a HER3 antibody, topoisomerase I inhibitor, with an ADC that consists of an exatecan derivative that is a topoisomerase I inhibitor (e.g., DS-8201) and with a govitecan derivative (e.g., IMMU-132). 6. Medical history of symptomatic congestive heart failure or serious cardiac arrhythmia requiring treatment; myocardial infarction within 6 months prior to enrolment or unstable angina. 7. QT interval corrected using Fridericia's formula to >450 ms in males and > 470 ms in females. 8. Any factor that increases the risk of corrected QT interval prolongation or risk of arrhythmic events, such as congenital long QT syndrome, family history of long QT syndrome, or unexplained sudden death under 40 years of age in first-degree relatives. 9. Medical history of clinically significant lung diseases or who are suspected to have these diseases by imaging at the screening period. 10. Clinically significant corneal disease. 11. Known hypersensitivity to either the drug substance components or inactive ingredients in the drug product or history of severe hypersensitivity reactions to other monoclonal antibodies. 12. Clinically severe pulmonary compromise resulting from intercurrent pulmonary illnesses including, but not limited to, any underlying pulmonary disorder and any autoimmune, connective tissue, or inflammatory disorders with potential pulmonary involvement or prior pneumonectomy.

All patients will undergo pretreatment tumor tissue acquisition. Central determination of *ERBB3* mRNA expression will be performed in FFPE core biopsies, and patients will be enrolled in four cohorts, according to the expression of *ERBB3* based in quartiles and defined by the pre-specified cutoffs, to ensure a broad representation of HR-positive/HER2-negative tumors with different *ERBB3* expression. The number of slots available per cohort will be limited to 20 patients each.

After confirmation of all the eligibility criteria, patients will be enrolled, and a single dose of patritumab deruxtecan will be administered by intravenous infusion at a dose of 6.4 mg/kg. A second optional biopsy will be performed in the same lesion 3–7 days after patritumab deruxtecan's administration. A third biopsy post-treatment of the same lesion will be mandatory 21 (±3) days after the administration of patritumab deruxtecan, independently of the subsequent treatment. Thereafter, patients will be considered either for definitive surgery or primary medical treatment (e.g., neoadjuvant chemotherapy) at the discretion of the treating physician.

### Primary Endpoint—The CelTIL Score

To answer the primary objective of the trial, we will evaluate CelTIL score differences between baseline and post-treatment samples in all patients regardless of their *ERBB3* mRNA expression. The CelTIL score is based on the percentage (%) of tumor cellularity and the % of stromal TILs. Histopathologic analysis of the proportion of TILs will be done in whole sections of tumor tissue stained with hematoxylin and eosin (H&E). TILs will be quantified according to the 2014 guidelines developed by the International TILs Working Group (25). Percentages of TILs and tumor cellularity at baseline and D21 will be scored in slides of core biopsies from patients enrolled in the trial blinded from clinic–pathologic and outcome data.

The CelTIL score was developed based on day 15 tumor samples from the PAMELA trial (22). The neoadjuvant PAMELA trial treated 151 HER2+ breast cancer patients with trastuzumab-lapatinib (and endocrine therapy if HR-positive) (26). Tumor cellularity and the TILs score measured at day 15 following anti-HER2 therapy was associated with pathologic complete response (pCR). A combined score, CelTIL, considering both variables was derived: CelTIL score = −0.8 × tumor cellularity (in %) + 1.3 × TILs (in %). The CelTIL score was validated in the PAMELA (26) and LPT109096 (27) phase II neoadjuvant trials as an early readout of the probability of a pCR. High CelTIL scores identify tumors that have high immune infiltration and reduced tumor cellularity (22).

In a third study, the CelTIL score was performed in tumor samples of 196 patients with early-stage HER2-positive disease treated with standard trastuzumab-based chemotherapy from the NeoALTTO phase III trial (28). This study randomized 455 women with HER2-positive early breast cancer to lapatinib (Arm A), trastuzumab (Arm B), or trastuzumab and lapatinib (Arm C) for 6 weeks, followed by an assigned anti-HER2 treatment combined with paclitaxel weekly. The CelTIL score was independently associated with event free survival, overall survival, and pCR (29). Early and absolute changes in the CelTIL score following neoadjuvant therapy were associated with tumor shrinkage at surgery in other three neoadjuvant trials (30). Taken together, these results demonstrated that high TILs and low tumor cellularity following one cycle of treatment provided independent and additional predictive information in patients with primary breast cancer following neoadjuvant treatment, also suggesting that CelTIL could be a surrogate for treatment efficacy in the neoadjuvant setting.

Secondary endpoints, summarized in Table 2, include mean change in the CelTIL score in ultralow, low, medium, and high ERBB3 cohorts, correlation between *ERBB3* mRNA and HER3 IHC baseline levels and changes in the CelTIL score, the CelTIL score according to PAM50 intrinsic subtype, antiproliferative activity, and safety. Exploratory and translational research endpoints include the assessment of predictive and prognostic biomarkers.

**Table 2:** Primary and secondary objectives and endpoints.

**Primary objective**	**Primary endpoint**
To evaluate if one dose of U3-1402 increases the value of the CelTIL score between baseline and post-treatment samples in all included patients with early breast cancer.	Mean change in the CelTIL score per central assessment in paired samples after one dose of U3-1402 at C1D21 (±3). CelTIL score = −0.8 × tumor cellularity (in %) + 1.3 × TILs (in %). The minimum and maximum unscaled CelTIL scores will be −80 and 130. This unscaled CelTIL score will then be scaled to reflect a range from 0 to 100 points.
**Secondary objectives**	**Secondary endpoints**
To identify a significant increase in the CelTIL score after one dose of U3-1402 between baseline and post-treatment samples within each of the four *ERBB3* cohorts.	Mean change in the CelTIL score at C1D21 of treatment in paired samples in ultralow, low, medium, and high *ERBB3* cohorts.
To determine the association of the levels of baseline *ERBB3* expression with changes in the CelTIL score after one dose of U3-1402 in all patients and within each ERBB3 cohort.	Correlation between *ERBB3* mRNA baseline levels and changes in the CelTIL score at C1D21 in paired samples in all patients and in ultralow, low, medium, and high ERBB3 cohorts.
To determine the association of HER3 IHC expression with changes in the CelTIL score after a single dose of U3-1402 in all patients and within each *ERBB3* cohort.	Correlation between HER3 IHC levels per central assessment and changes in the CelTIL score at C1D21 in paired samples in all patients and in ultralow, low, medium, and high *ERBB3* cohorts.
To evaluate the changes in CelTIL across the four PAM50 intrinsic subtypes.	CelTIL score at the C1D21 score according to intrinsic subtype: Luminal A, Luminal B, HER2-enriched, and Basal-like subtypes.
To evaluate the antiproliferative activity of one dose of U3-1402 between baseline and post-treatment samples.	Complete Cell Cycle Arrest (CCCA) determined per central assessment by IHC Ki67 <2.7% at C1D21.
	Differences in differential expression [mean suppression = 100–[geometric mean (post-treatment/pre-treatment 100)]] of proliferative genes (*BIRC5, CCNB1, CDC20, CDCA1, CEP55, KNTC2, MKI67, PTTG1, RRM2, TYMS*, and *UBE2C*).
To evaluate the association of *ERBB3* mRNA expression with HER3 IHC expression.	Correlation coefficients between both biomarkers.
To evaluate the changes of HER3 expression.	HER3 IHC at baseline, at D3-D7 (optional), C1D21.
To describe the safety and tolerability of U3-1402.	Type, incidence, severity (as graded by the NCI CTCAE v. 5.0), seriousness, and attribution to the study medications of AEs and any laboratory abnormalities.

### Measuring *ERBB3* mRNA

Each patient will be assigned to one of the four cohorts according to their *ERBB3* mRNA expression in the baseline sample determined by the nCounter Platform. The cutoffs to be used in this trial were determined as follows.

To date, we have analyzed *ERBB3* mRNA using the nCounter platform in 1,600 tumor samples using formalin-fixed paraffin-embedded tumor samples with IHC data. Among these samples with IHC data, 65% were HR-positive and 18% were HER2-positive. The IHC subtype distribution is as follows: (1) 51.9% HR-positive/HER2-negative, (2) 29.9% triple-negative breast cancer (TNBC), (3) 13.5% HR-positive/HER2-positive, and (4) 4.7% HR-negative/HER2-positive.

In this nCounter dataset, the range of *ERBB3* mRNA expression has an 18.6-fold difference in gene expression (i.e., from the lowest to the highest *ERBB3* value), and the interquartile range is 1.5 (in log base 2), which is equal to a difference in expression of 2.9-fold.

**Figure 2:**
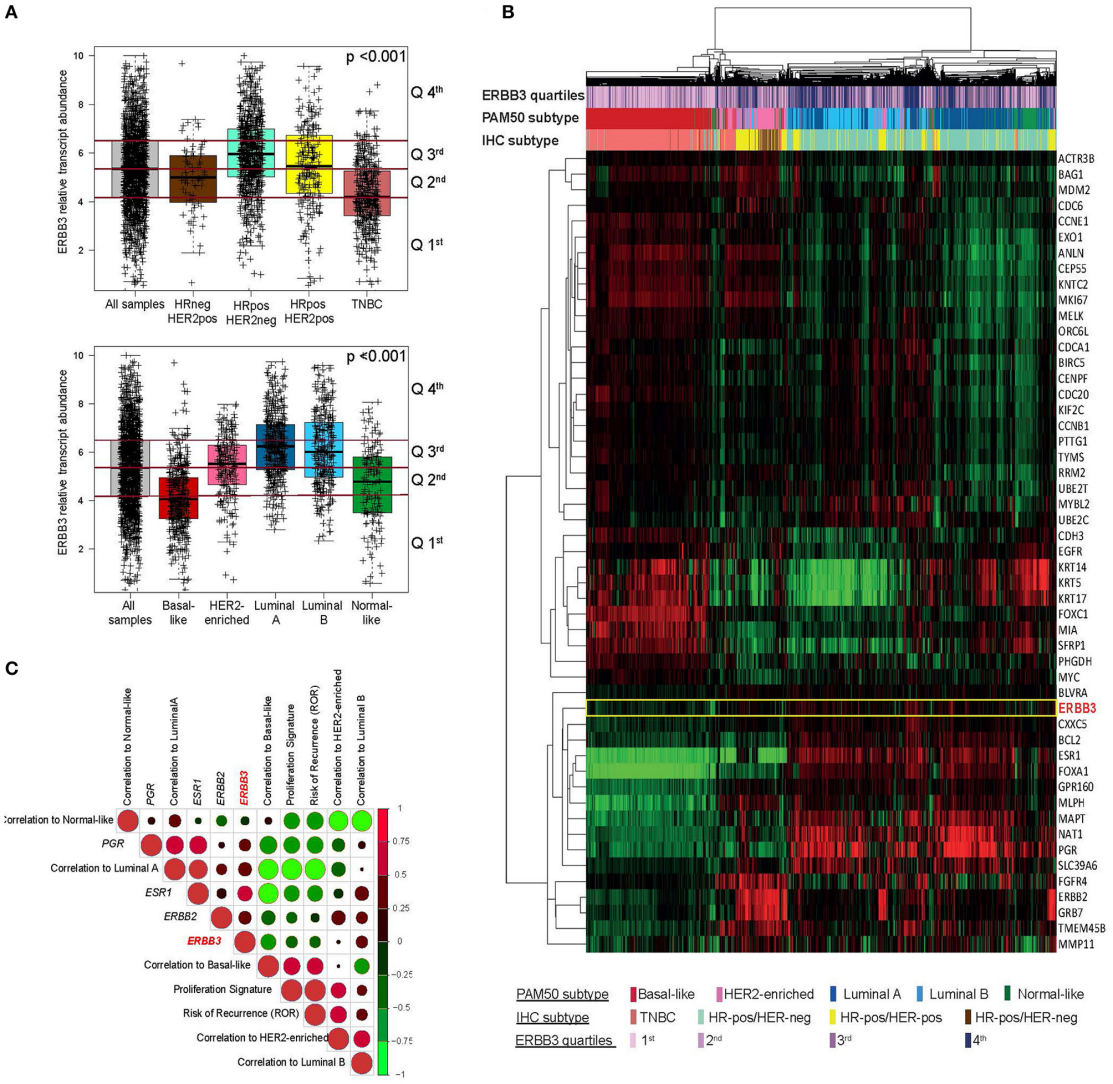
Measurement of ERBB3 expression in breast cancer using the nCounter platform. **(A)** Box plots of *ERBB3* gene expression in breast tumors as classified by hormone receptor and *HER2* expression and intrinsic subtype. **(B)** Unsupervised hierarchical clustering using the 50 PAM50 genes and *ERBB3* (rows) and 1,580 tumor samples (columns). Each colored square on the heatmap represents the relative median signature score for each sample with the highest expression being red, the lowest expression being green, and the average expression being black. **(C)** Pearson correlation between *ERBB3*, single genes, and PAM50 gene expression signatures evaluated in breast cancer samples.

Large expression variability across and within each IHC-based and PAM50 subtype was observed. *ERBB3* expression was statistically significantly higher in HR-positive tumors (*P* < 0.001; Figure 2A). *ERBB3* expression varied statistically significantly according to the intrinsic subtype (*P* < 0.001; Figure 2A), with the Luminal A subtypes showing the highest median expression, followed by the Luminal B, HER2-enriched, and Basal-like.

Using quartiles, the proportion of *ERBB3*-high tumors within each IHC subtype ranged from 4% in TNBC to 36% in HR+/HER2-negative when percentile 75th in the combined matrix was used as the cutoff to define *ERBB3*-high (Figure 2A).

Next, we explored the association of *ERBB3* expression with PAM50 breast cancer-related genes in the combined matrix (Figure 2B). As expected, *ERBB3* high correlated [correlation coefficients [r] > 0.50] with a group of five genes, including *ESR1* and *FOXA1*, which are significantly enriched in luminal and hormone response biology. Concordant with this single-gene analysis, moderate correlation (r = 0.53) was found between *ERBB3* and PAM50 Luminal A signature and negative correlation (r = −0.25) between *ERBB3* and PAM50 Basal-like, proliferation, and risk of recurrence signatures (Figure 2C).

### Evaluating *ERBB3* Expression in Independent Datasets

**Figure 3:**
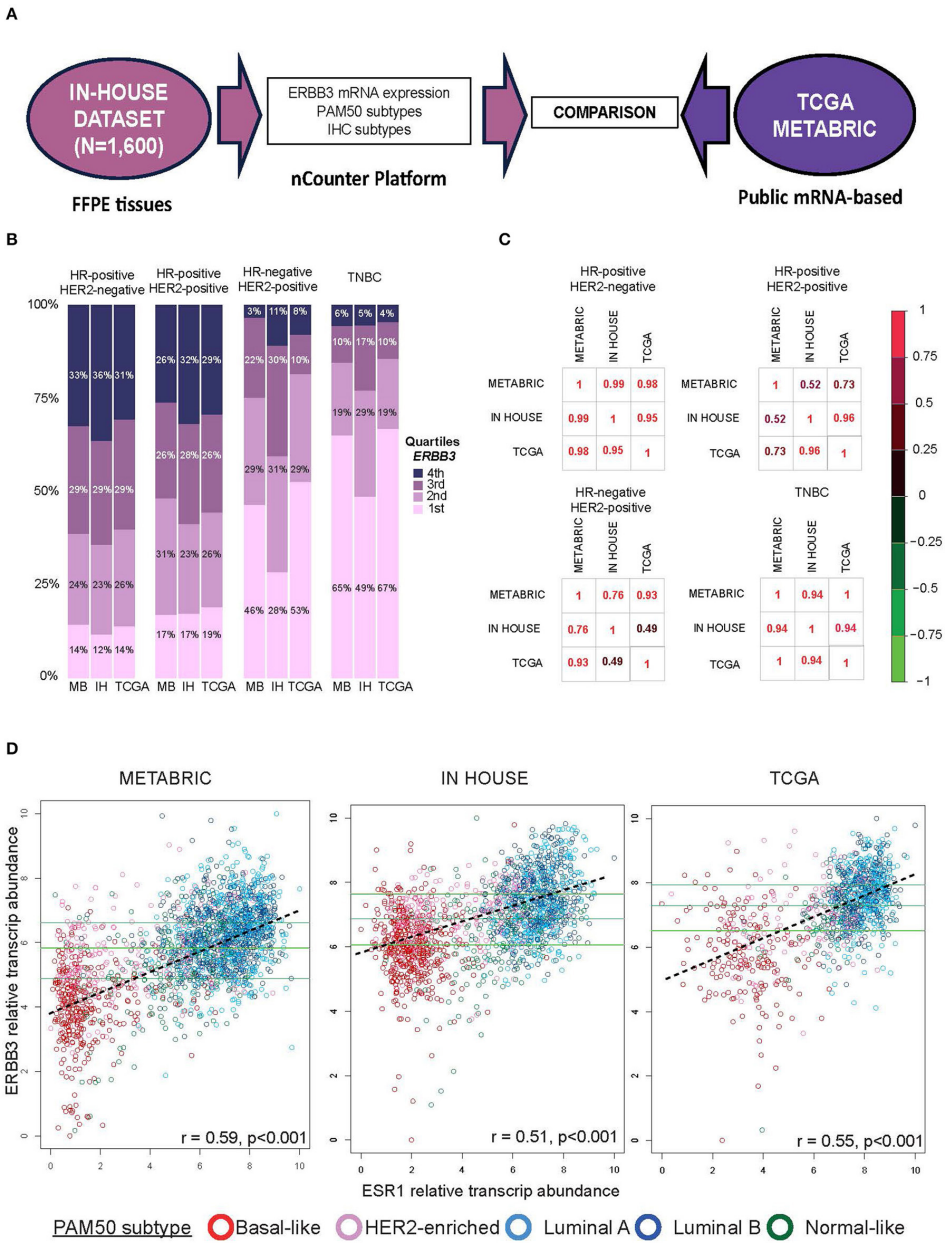
Comparing *ERBB3* expression across datasets **(A)** Evaluation of *ERBB3* cutoff in breast cancer samples from patients with early breast cancer included in IN-HOUSE, METABRIC, and TCGA. **(B)** Proportion of samples in each immunohistochemistry subtype based on the ERBB3 cohort. Each bar is colored according to the ERBB3 distribution in each cohort. **(C)** Correlation coefficients of proportions of tumor samples within each quartile based on the IHC subtype between the three datasets. **(D)** Scatter plots of *ERBB3* vs. *ESR1* expression for samples from METABRIC, IN-HOUSE, and TCGA cohorts, colored by subtype. The three horizontal lines indicate the cutoffs of each cohort. Discontinued line in each figure represents the regression line. Pearson correlation coefficient (r) with significance (*p-*value) is presented in each figure.

In order to examine the consistency of the cutoff points, results from the in-house nCounter dataset were compared to two independent cohorts (i.e., METABRIC and TCGA datasets). METABRIC includes 1,992 breast cancer samples analyzed by the Illumina HT 12 IDATS platform, and TCGA includes 1,101 breast cancer samples analyzed by HiSeq Illumina sequencers (Figure 3A).

Using quartiles, Figure 3B shows the proportion of tumors within each quartile based on their IHC subtype between our in-house dataset, METABRIC, and TCGA. Figure 3C shows the correlation coefficients among the three datasets in the different IHC-group tumors. In HR-positive/HER2-negative, the correlation coefficients of the proportions between the three datasets were remarkably similar. In the other subtypes, the correlation coefficients among the datasets were between 0.49 and 0.99. A relationship between *ERBB3* and *ESR1* expression was seen to be moderately correlated across the three datasets (Figure 3D); the correlation coefficients among the datasets were between 0.51 and 0.59.

### Statistical Analysis

The study would require a sample size of 72 (number of pairs samples) to achieve a power of 80% using a level of significance of 5% (two sided), for detecting a mean difference between pairs of 13 CelTIL score. It is assumed that the standard deviation of the differences is 38.6, which is the standard deviation observed in 403 patients with CelTIL data across the four SOLTI trials (30). Assuming a 10% drop-out or lack of available tissue, 80 patients will be recruited.

No formal statistical comparison will be carried out between cohorts. Statistical analyses will be performed to estimate the proportions or means (or medians) for all variables including confidence interval calculations.

## Conclusion

We propose the TOT-HER3 study, the first window of opportunity trial to evaluate the biological effect of patritumab deruxtecan in patients with HR-positive/HER2-negative early breast cancer. High *ERBB3* mRNA gene expression is observed across all subtypes of breast cancer, although it predominates in HR-positive/HER2-negative disease suggesting a role for HER3 directed therapies in this disease. We will analyze *ERBB3* expression using a clinically applicable assay in FFPE primary tumors.

This information can provide insight for improving the design of future clinical trials in the HR-positive/HER2-negative breast cancer through the selection of patients who will mostly benefit from this drug. The use of a quantitative method such as *ERBB3* mRNA expression, which offers the opportunity to identify different cutoffs, might potentially improve treatment personalization. In addition, the results of TOT-HER3 could help identify patients most likely to benefit from HER3 directed ADCs across cancer types.

## Data Availability Statement

Data from Breast tumor samples with available RNASeqv2 data at the TCGA portal was downloaded. Metabric expression data are available at the European Genome-Phenome Archive (https://ega-archive.org/), which is hosted by the European Bioinformatics Institute, under accession number EGAS00000000083. The rest of the data are available upon reasonable request.

## Author Contributions

All authors participated in the design and/or interpretation of the reported results and participated in the acquisition and/or analysis of data. In addition, all authors participated in drafting and/or revising the manuscript and provided administrative, technical, or supervisory support.

## Conflict of Interest

SE and PF are employed by Daiichi Sankyo, Inc. AP has declared personal honoraria from Pfizer, Novartis, Roche, MSD Oncology, Lilly, and Daiichi Sankyo, travel, accommodations, and expenses paid by Daiichi Sankyo, research funding from Nanostring Technologies, Roche, and Novartis, and consulting/advisory role for Nanostring Technologies, Roche, Novartis, Pfizer, Oncolytics Biotech, Amgen, Lilly, MSD, PUMA, and Daiichi Sankyo, Inc. outside the submitted work. MO reports honoraria and consulting fees from Roche/Genentech, GSK, PUMA Biotechnology, AstraZeneca, Seattle Genetics, and Novartis; travel and accommodation paid by Roche, Pierre-Fabre, Novartis, GP Pharma, Grünenthal, and Eisai; and grant/Research Support (to the Institution) from AstraZeneca, Philips Healthcare, Genentech, Roche, Novartis, Immunomedics, Seattle Genetics, GSK, Boehringer-Ingelheim, PUMA Biotechnology, and Zenith Epigenetics outside the submitted work. EC reports personal fees from Roche, personal fees from Lilly, personal fees from Novartis, and personal fees from Pfizer, during the conduct of the study. SP reports an advisor/consultant role for AstraZeneca, Daiichi-Sankyo, Polyphor, and Roche, and travel and accommodation paid by Novartis. JC reports an advisor/consultant role for Roche, Novartis, Pfyzer, Pharmamar, Lilly, Eisai, and Amgen, and travel and accommodation by Novartis and Pharmamar. FS-B has declared personal honoraria from Pfizer, Novartis, Roche, and Daiichi Sankyo. PV has received honoraria as a Speaker from Nanostring. MM has declared an advisor role or consulting from Novartis, Pfizer, and Roche; research funding from Roche, Eisai, and AstraZeneca; and travel expenses from Roche. OM-S reports an advisor role from Roche; honoraria as a speaker from Eisai; and travel expenses from Novartis. GV reports receiving honoraria for speaker activities from MSD and an advisory role from AstraZeneca. CS has served as a consultant, participated in advisory boards, or received travel grants from AstraZeneca, Celgene, Daiichi Sankyo, Eisai, F. Hoffmann—La Roche Ltd., Genomic Health, Merck, Sharp and Dhome España S.A., Novartis, Odonate Therapeutics, Pfizer, Philips Healthwork, Pierre Fabre, prIME Oncology, Puma, Synthon, and Sanofi Aventis. The remaining authors declare that the research was conducted in the absence of any commercial or financial relationships that could be construed as a potential conflict of interest.
